# EHMTI-0203. Increased prevalence of migraine without aura in patients with saccular intracranial aneurysms (SIA) and remission after clipping of the aneurysm

**DOI:** 10.1186/1129-2377-15-S1-D36

**Published:** 2014-09-18

**Authors:** ER Lebedeva, AV Busygina, EY Guzhina, VS Kolotvinov, VP Sakovich, J Olesen

**Affiliations:** 1 Neurology, the Urals state medical university International Headache Center "Europe-Asia", Yekaterinburg, Russia; 2 Neurology and Neurosurgery, the Urals state medical university, Yekaterinburg, Russia; 3 Neurosurgery, City Hospital 40 Regional neurosurgical center, Yekaterinburg, Russia; 4 Neurology, Glostrup Hospital University of Copenhagen, Copenhagen, Denmark

## Introduction

The aim of our study was to determine 1-year prevalence of headache before rupture of saccular intracranial aneurysms (SIA) and 1-year after clipping of SIA.

## Methods

We prospectively studied 199 consecutive patients with SIA (103 females and 96 males, mean age: 43.2 years).As control served 194 blood donors (86 females, 108 males, mean age: 38.4 years). Both groups were interviewed about headaches in the year preceding clipping/interview using a validated semi-structured neurologist conducted interview. SIA patients with a past history of recurrent headaches (N=87) were follow-up 1 year after clipping. The remission rates of migraine and tension-type headache (TTH) in these patients was compared to 92 patients from a headache center interviewed twice one year apart. Diagnoses were made according to the ICHD-2.

## Results

During the year before rupture, 124(62.3%) had one or more types of headache: migraine without aura (MO) -78(39.2%), migraine with aura - 2(1%), probable migraine: 4(2%), TTH: 39(19.6%), cluster headache: 2(1%).Only the prevalence of MO was significantly higher in patients with SIA compare to controls (39.2% VS 8.8%, OR 6.7, 95% CI 3.8-11.9, p<0.0001).One year after clipping, the prevalence of MO was significantly more reduced in patients with SIA than in controls (74.5% VS 12.8%, p<0.0001).No factors except clipping of the aneurysm could explain the remission of migraine. The prevalence of TTH did not change significantly after clipping of SIA but decreased significantly after treatment in controls.

## Conclusions

Unruptured SIA is associated with a marked increase in the prevalence of migraine without aura which decreases significantly after clipping.

No conflict of interest.

